# Oxidative Stress and Endothelial Dysfunction in Patients with Hypertensive Nephropathy: Role of the Mediterranean Diet

**DOI:** 10.3390/ijms27031320

**Published:** 2026-01-28

**Authors:** Luca Salomone, Danilo Menichelli, Irene Azzara, Pierluigi Maria Damosso, Vittoria Cammisotto, Valentina Castellani, Pasquale Pignatelli, Elena Pacella, Anna Paola Mitterhofer, Francesca Tinti, Silvia Lai

**Affiliations:** 1 Nephrology Unit, Department of Translational and Precision Medicine, Sapienza University of Rome, 00185 Rome, Italy; ireneazzara@gmail.com (I.A.); silvia.lai@uniroma1.it (S.L.); 2 Department of General Surgery, Surgical Specialty and Anesthesiology, Sapienza University of Rome, 00185 Rome, Italy; danilo.menichelli@uniroma1.it; 3 Ophthalmology Unit, Department of Sense Organs, Sapienza University of Rome, 00185 Rome, Italy; pierluigimaria.damosso@uniroma1.it (P.M.D.); elena.pacella@uniroma1.it (E.P.); 4 Department of Clinical Internal, Anesthesiological and Cardiovascular Sciences, Sapienza University of Rome, 00185 Rome, Italy; vittoria.cammisotto@uniroma1.it (V.C.); valentina.castellani@uniroma1.it (V.C.); pasquale.pignatelli@uniroma1.it (P.P.); 5 Nephrology Unit, Department of Systems Medicine, Tor Vergata Universisty of Rome, 00133 Rome, Italy; annapaola.mitter@ptvonline.it; 6 Nephrology Unit, Department of Molecular Medicine, Sapienza University of Rome, 00185 Rome, Italy

**Keywords:** Med-Diet, oxidative stress, endothelial dysfunction, hypertensive nephropathy

## Abstract

Essential hypertension is a leading cause of chronic kidney disease (CKD) and is frequently complicated by hypertensive nephropathy, characterized by nephroangiosclerosis and increased intrarenal vascular resistance, assessable by renal resistive index (RRI). Oxidative stress and endothelial dysfunction contribute to CKD progression, and the Mediterranean diet (MD) has been associated with a more favorable oxidative and endothelial profile, although data linking diet to renal microcirculation in hypertensive nephropathy remain limited. The aim of this study is to evaluate the relationship between RRI, oxidative stress, endothelial function, and adherence to the Mediterranean diet in patients with essential hypertension and hypertensive nephropathy. We performed a cross-sectional single-center study and we enrolled 99 patients with essential hypertension, hypertensive nephropathy, and CKD stages G1–G4 (KDIGO). All patients underwent laboratory testing, measurement of oxidative stress markers (sNOX2-dp, H_2_O_2_) and endothelial function (NO), renal ultrasound with interlobar RRI assessment, and PREDIMED questionnaire for MD adherence. A significant direct correlation was observed between RRI and oxidative stress markers (sNOX2-dp and H_2_O_2_) (*p* = 0.002, r = 0.302; *p* = 0.002, r = 0.322), while a significant inverse correlation was found between RRI and the endothelial function marker (NO) (*p* = 0.013, r = −0.302). The correlation between RRI and PREDIMED questionnaire scores did not reach statistical significance, but there was a trend toward an inverse association (*p* = 0.06, r = −0.18). In addition, a significant inverse correlation was observed between RRI and eGFR (*p* = 0.005, r = −0.27), consistent with published data. We also found a significant inverse correlation between sNOX2-dp and PREDIMED scores (*p* = 0.034, r = −0.21); no statistically significant correlations with H_2_O_2_ and NO were observed in this analysis. Higher intrarenal vascular resistance is associated with heightened oxidative stress, impaired endothelial function, and lower eGFR. Adherence to the Mediterranean diet is linked to lower NOX2-mediated oxidative stress, supporting a potential association between higher MD adherence and lower NOX2-related oxidative stress. These findings are hypothesis-generating and require confirmation in adequately powered longitudinal and interventional studies before any clinical inference on CKD progression can be made.

## 1. Introduction

Essential arterial hypertension is an extremely widespread condition globally and is constantly increasing; it is estimated to affect over one billion adults worldwide [1,2]. Arterial hypertension is one of the main causal factors of target organ damage, especially the affecting renal and cardiovascular systems. Hypertensive nephroangiosclerosis is counted among the leading causes of chronic kidney disease (CKD) worldwide [3]. Hypertensive patients with renal involvement have a markedly higher cardiovascular risk, and CKD contributes to the increased cardiovascular mortality observed in individuals with uncontrolled hypertension [1]. Several mechanisms were involved in the pathogenesis of kidney and cardiovascular injury related to arterial hypertension. In particular, oxidative stress and endothelial dysfunction are known to be more pronounced in patients with essential arterial hypertension [4]. Indeed, arterial hypertension involves several molecular mechanisms as hyperactivation of the renin–angiotensin–aldosterone system leading to a chronic inflammatory state and an excessive production of reactive oxygen species (ROS) [4]. The excess ROS results in reduced bioavailability of nitric oxide (NO), with consequent vasoconstriction and vascular remodeling [4].

Moreover, in patients with hypertensive nephropathy, the decline in the glomerular filtration rate and the accumulation of uremic toxins further amplify the oxidative imbalance and contribute to systemic endothelial dysfunction [5]. This creates a vicious circle in which oxidative stress and endothelial dysfunction mutually reinforce each other, worsening renal hypoxia and fibrosis and increasing the risk of cardiovascular complications in patients with hypertensive nephropathy.

Several signs and measures were tested to assess early subclinical injury of kidney vascularization. One of the most validated and used indexes worldwide is renal resistive index (RRI). Indeed, in patients with arterial hypertension, RRI assessment provides useful information on target organ damage and on kidney vascularization [6]. It has been evidenced several times that, in patients with essential arterial hypertension, an RRI > 0.7 is a subclinical sign of target organ damage and correlates with a faster progression of chronic kidney disease [6].

In this context, the Mediterranean diet (MD) is a dietary pattern that has shown efficacy in improving several cardiovascular risk factors: in primary prevention, compared with other dietary regimens, the MD is associated with lower blood pressure values and a more favorable lipid profile [7]. Furthermore, large randomized clinical trials have demonstrated a reduction in major cardiovascular events in high-risk individuals with high adherence to MD [7]. In particular, the PREDIMED trial showed that intensive adherence to the MD leads to an improvement in endothelial function in subjects at high cardiovascular risk [8,9].

However, the link between oxidative stress and endothelial dysfunction and the worsening of RRI in patients with hypertensive nephropathy remains poorly explored as well as the role of MD on the oxidative stress and endothelial disfunction imbalance. For this reason, the aim of this study was to investigate the role of oxidative stress and endothelial dysfunction markers on subclinical organ injury as RRI in patients with hypertensive nephropathy, and to assess the role of the MD on oxidative stress and endothelial dysfunction imbalance.

## 2. Results

Overall, 99 patients were analyzed and divided into two groups according to renal resistive index (RRI) percentiles: 71 subjects with an RRI < 75th percentile and 28 with an RRI ≥ 75th percentile (Table 1). In patients with an RRI ≥ 75th percentile, median age was higher than in those with an RRI < 75th percentile, whereas no significant differences were observed in terms of BMI, systolic, diastolic, or mean blood pressure. Mean eGFR was lower in the RRI ≥ 75th percentile group (69.9 ± 21.0 vs. 79.1 ± 19.4 mL/min/1.73 m^2^), while serum creatinine was 0.98 ± 0.04 vs. 1.09 ± 0.08 mg/dL. The distribution by sex and smoking status was similar between the two groups, whereas the prevalence of dyslipidemia was higher in the RRI ≥ 75th percentile group. Use of diuretics or calcium-channel blockers was comparable between groups, while treatment with β-blockers and statins was more frequent in the RRI ≥ 75th percentile group. With regard to biomarkers, NO levels were lower and H_2_O_2_ levels higher in the RRI ≥ 75th percentile group, whereas sNOX2-dp values did not significant differ between two groups.

**Figure 1:**
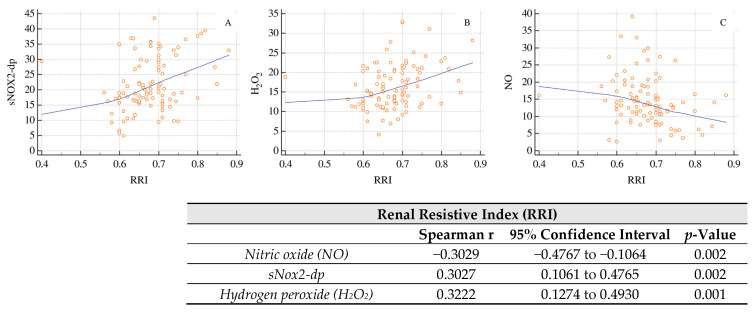
Correlations between renal resistive index (RRI), oxidative stress, and endothelial dysfunction biomarkers ((Panel A): sNOX2-dp, (Panel B): hydrogen peroxide, (Panel C): Nitric oxide).

**Figure 2:**
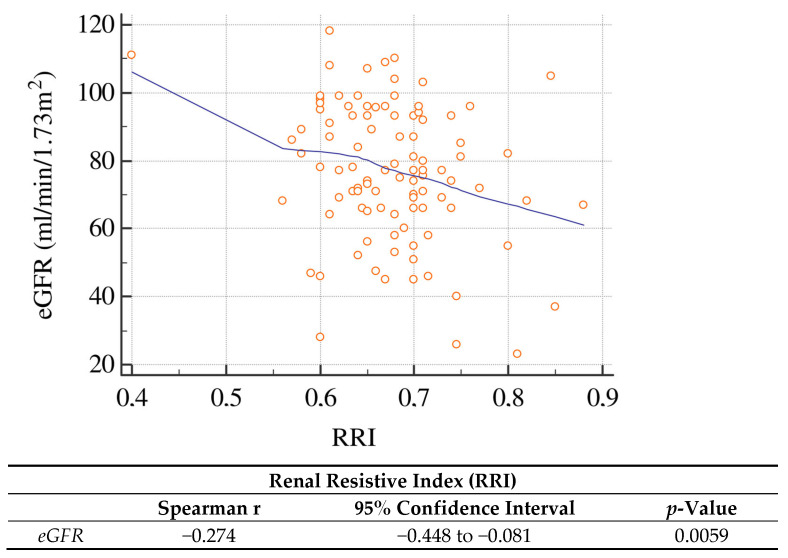
Correlation between renal resistive index (RRI) and eGFR.

Figure 1 shows the correlations between RRI and biomarkers of oxidative stress and endothelial dysfunction. RRI displays a statistically significant negative correlation with NO levels (Spearman r = −0.3029; *p* = 0.002) and statistically significant positive correlations with sNOX2-dp (r = 0.3027; *p* = 0.002) and H_2_O_2_ (r = 0.3222; *p* = 0.001). RRI was also inversely correlated with eGFR as shown in Figure 2 (*p* = 0.0059).

Furthermore, we performed a multivariable stepwise linear regression analysis (Table 2) to evaluate the association between RRI and Ox stress biomarkers adjusting for potential confounding factors as age, sex, and use of beta-blockers and statins. We found a significant direct association between RRI and sNOX2-dp and H_2_O_2_ serum levels and an inverse association between RRI and NO serum levels (Table 2).

**Figure 3:**
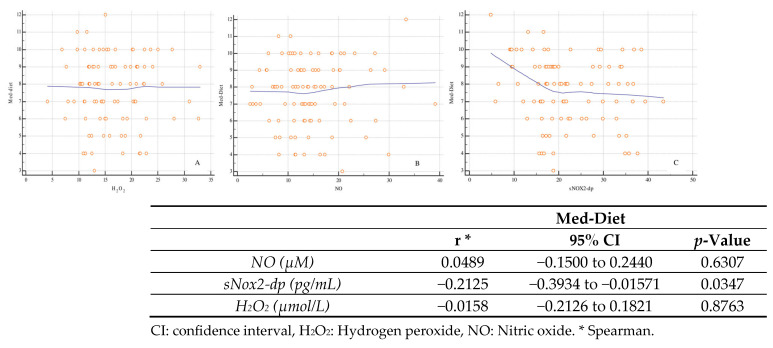
Correlations between Mediterranean diet (Med-Diet) and oxidative stress and endothelial function indices ((**Panel A**): hydrogen peroxide, (**Panel B**): Nitric Oxide, (**Panel C**): sNOX2-dp).

Figure 3 illustrates the correlations between the Mediterranean diet adherence score (Med-Diet) and biomarkers of oxidative stress and endothelial function. The only inverse correlation reaching statistical significance is that with sNOX2-dp (r = −0.2125; *p* = 0.0347); an inverse correlation is also observed for H_2_O_2_, although it does not reach statistical significance (r = −0.0158; *p* = 0.8763), while a direct correlation is seen for NO (r = 0.0489; *p* = 0.6307), which is likewise not significant.

## 3. Discussion

The data from our cross-sectional study show a significant correlation between the RRI, oxidative stress and endothelial function in patients with essential arterial hypertension and hypertensive nephropathy. We found a significant difference in sNOX2-dp, H_2_O_2_ and NO levels, with a direct correlation between RRI and oxidative stress markers (sNOX2-dp, H_2_O_2_) and an inverse correlation between RRI and markers of endothelial dysfunction (NO).

These findings are coherent with evidence indicating that RRIs are indicators of subclinical renal vascular damage and of a potential increase in cardiovascular risk in hypertensive patients. Studies conducted in hypertensive cohorts have indeed shown that an increase in RRI is associated with early signs of target-organ damage (albuminuria, left ventricular hypertrophy, increased aortic stiffness) and may reflect subclinical intrarenal atherosclerosis [10]. More recent reviews confirm that RRI, measured in interlobar arteries, is a marker of renal vascular and interstitial damage, and that higher values are associated with an increased risk of CKD progression and cardiovascular events [11]. In this context, the association that we observed between RRI and markers of oxidative stress/endothelial function suggests that increased intrarenal resistance may be one of the peripheral manifestations of a systemic activation of pro-oxidant pathways and endothelial dysfunction.

It is well known that, from a pathophysiological standpoint, essential hypertension is closely linked to increased oxidative stress and reduced NO bioavailability. It is well documented that, in the arteries of hypertensive patients, there is an increased production of ROS by NADPH oxidase and other pro-oxidant enzymes, resulting in NO inactivation and impaired endothelium-dependent vasodilation [4]. In this setting, NOX2 represents one of the main NADPH oxidase isoforms involved in hypertensive vascular damage, and the soluble fragment sNOX2-dp has been validated as a circulating marker of NOX2 activation and systemic oxidative stress in various cardiovascular conditions [12]. Our finding of higher sNOX2-dp and H_2_O_2_ serum levels and concomitant lower serum levels of NO in subjects with higher RRI is coherent with this model: more intense activation of oxidative pathways may promote vasoconstriction, vascular remodeling, and increased intrarenal resistance, which is reflected in higher RRI values.

A further point of interest is the inverse relationship correlation between eGFR and RRI. In our cohort, a reduction in the glomerular filtration rate is accompanied by a significant increase in RRI, suggesting that the decline in renal function is associated with progressive impairment of renal parenchymal perfusion. Observational studies in patients with non-diabetic CKD and other nephropathies have shown that higher RRI values predict a more rapid decline in eGFR and higher mortality, even independently of proteinuria [13]. Recent systematic reviews confirm that RRI is an important diagnostic and prognostic parameter in the assessment of patients with CKD, being associated with an increased risk of progression to advanced stages of renal failure and of cardiovascular events [14]. In patients with long-standing arterial hypertension, nephroangiosclerosis leads to arteriolar wall thickening, capillary rarefaction and interstitial fibrosis, with increased intrarenal resistance and reduced cortical blood flow; these structural changes are likely to underlie both the increase in RRI, and the progressive loss of renal function described in the literature [11]. Our data therefore fit within this framework, showing that in subjects with hypertensive nephropathy, worsening glomerular filtration, increased RRI, and activation of oxidative stress tend to coexist.

Regarding the Mediterranean diet, in our study we observed a significant inverse association between adherence to the Mediterranean pattern and sNOX2-dp levels, whereas the correlations with NO and H_2_O_2_ did not reach statistical significance, although they remained consistent with the hypothesis of an antioxidant effect of the diet. Notably, the association between MD adherence and RRI did not reach conventional statistical significance and should therefore be interpreted as a trend-level finding rather than supportive evidence of a diet–microcirculation effect. These findings are in line with observational and interventional studies showing that greater adherence to the Mediterranean diet is associated with lower levels of oxidative stress markers and better endothelial function. A systematic review of studies on dietary patterns and oxidative stress biomarkers reported that Mediterranean-type diets are associated with a reduction in several indicators of oxidative damage [15]. Randomized trials and meta-analyses have also shown that Mediterranean diet–based interventions can lower blood pressure and improve endothelium-dependent vasodilation, both in healthy subjects and in patients at high cardiovascular risk [16].

Specifically regarding sNOX2-dp, studies in patients with cardiovascular disease or metabolic syndromes have shown that greater adherence to the MD is associated with lower sNOX2-dp levels and other oxidative stress markers, with a gradual reduction in sNOX2-dp values from low to high MD scores [17,18]. A review of dietary patterns confirms that the Mediterranean diet is one of the nutritional models most consistently associated with a reduction in markers of NOX2 activation and circulating ROS [15]. The fact that, in our cohort, the statistically significant association emerges more clearly for sNOX2-dp than for NO and H_2_O_2_ may reflect greater sensitivity to this marker when detecting chronic changes in oxidative status related to dietary habits, as already hypothesized in other populations [12]. At the same time, the lack of statistical significance for the correlations with NO and H_2_O_2_ may depend on the relatively limited sample size, the biological variability of these parameters, and the absence of repeated measurements over time.

Overall, our findings are consistent with a potential link between dietary pattern, systemic redox status, and renal vascular damage: in patients with hypertensive nephropathy, high NOX2 activation is associated with high RRI and poorer endothelial function; conversely, greater adherence to the MD is associated with lower sNOX2-dp levels and, presumably, a reduction in systemic oxidative burden. Experimental and clinical studies support the hypothesis that the MD may modulate NADPH oxidase activity, improve NO bioavailability and attenuate vascular remodeling, with potentially favorable effects on the renal microcirculation as well [19]. However, given the cross-sectional design, these associations should be interpreted as hypothesis-generating rather than causal. In particular, any mechanistic inference that MD ‘modulates’ oxidative pathways or influences CKD progression cannot be supported by the present dataset and warrants confirmation in prospective studies.

Although sNOX2-dp is considered a sensitive marker of NOX2 activation, the absence of significant associations with NO and H_2_O_2_ should be interpreted cautiously. NO-related measures are characterized by substantial biological variability and are highly influenced by diet, renal function, inflammation, and pre-analytical factors (e.g., processing time and storage conditions), which may increase noise measurement in cross-sectional settings. Similarly, H_2_O_2_ levels may be affected by assay-related limitations and by its rapid turnover within antioxidant systems, resulting in transient fluctuations that a single measurement may fail to capture. Therefore, the differential behavior of these biomarkers likely reflects both biological kinetics and methodological constraints, and our results should be viewed as a composite redox profile rather than evidence driven by a single marker.

From a clinical standpoint, RRI can be obtained during routine renal Doppler ultrasonography with minimal additional burden and may help identify hypertensive CKD patients with higher intrarenal vascular resistance who could benefit from closer follow-up and intensified risk-factor control. Oxidative stress and endothelial biomarkers may complement RRI by providing a mechanistic ‘redox–vascular’ profile, particularly in patients with discordant clinical risk (e.g., relatively preserved eGFR but elevated vascular risk features). At present, these biomarkers should be regarded as investigational; however, they may inform hypothesis generation and patient selection for lifestyle or dietary interventions. Future research should include longitudinal studies assessing whether RRI and redox biomarkers predict renal and cardiovascular outcomes, as well as interventional trials evaluating whether structured Mediterranean diet programs can improve biomarker trajectories, RRI, and the rate of eGFR decline. Additional work is needed to establish assay reproducibility, clinically meaningful thresholds, and cost-effectiveness before routine implementation.

This study, however, has some limitations that should be considered when interpreting the results. First, the observational cross-sectional design, and the analysis based on a single assessment of RRI and biomarkers do not allow us to establish a causal relationship between increased RRI, oxidative stress and decline in renal function. Second, hypertensive nephropathy was defined clinically in the absence of histological confirmation. While extensive exclusion criteria were applied to reduce etiologic heterogeneity, some degree of diagnostic misclassification cannot be excluded, particularly regarding the exclusion of alternative causes of CKD (including proteinuric and primary glomerular diseases) in a real-world setting. Third, the sample size is relatively small and consists of patients followed at a single center, which may limit generalizability. In addition, RRI is also influenced by systemic hemodynamic factors (heart rate, pulse pressure) and by extra-renal vascular structural characteristics, as highlighted by methodological studies and recent reviews on the meaning of RRI [11]. Moreover, residual confounding cannot be excluded. Although we performed multivariable models for the association between RRI and biomarkers (adjusted for age, sex, and selected therapies), we could not comprehensively adjust all analyses for additional clinical covariates (e.g., dyslipidemia and other treatments) and lifestyle factors. Finally, dietary adherence was evaluated using a self-reported questionnaire collected at a single time point. This approach may be influenced by recall and social desirability biases and may not accurately capture long-term habitual dietary patterns or changes over time. Consequently, some degree of exposure misclassification cannot be excluded and may have biased associations toward the null, potentially contributing to the modest correlations observed. Future studies should consider repeated dietary assessments and/or complementary methods (e.g., dietary records and objective nutritional biomarkers) to better characterize long-term adherence.

In conclusion, our study shows that in patients with essential hypertension and hypertensive nephropathy, an increase in the RRI is correlated with a marked rise in oxidative stress markers (sNOX2-dp, H_2_O_2_) and a reduction in nitric oxide, suggesting a close link between renal perfusion, redox state, and endothelial function. The inverse correlation between eGFR and RRI confirms the value of this index as a potential integrated marker of renal vascular damage and disease progression. Moreover, the inverse relationship between adherence to the Mediterranean diet and sNOX2-dp levels supports the hypothesis that targeted nutritional interventions may help modulate oxidative stress in this population. Overall, our findings indicate that RRI and biomarkers of oxidative stress and endothelial function may represent useful tools for more refined risk stratification and for identifying patients who are potentially responsive to integrated therapeutic strategies, both pharmacological and dietary, to be confirmed in larger prospective and interventional studies.

## 4. Materials and Methods

### 4.1. Study Design and Subjects

We performed an observational, cross-sectional monocentric study on 99 consecutive Hypertensive Nephropathy patients at the University Hospital “Policlinico Umberto I” of Rome, Sapienza University of Rome, Italy. Patients were enrolled from April to July 2025. The study was approved by the local ethic committee of Sapienza University (No 7669 Prot. 0809/2024) and was conducted according to the 1975 Declaration of Helsinki. All patients signed informed written consent at study entry.

### 4.2. Inclusion Criteria

We recruited patients older than 18 years who had essential arterial hypertension diagnosed by 24 h ambulatory blood pressure monitoring (ABPM) and CKD stages G1–G4 with kidney damage secondary to arterial essential hypertension (eGFR calculated using the CKD-EPI equation in accordance with KDIGO guidelines) [20].

### 4.3. Exclusion Criteria

Patients with an age over 75 years, or CKD stage G5 or had undergone renal replacement therapy were excluded from this study. Furthermore, we also excluded patients with a history of diabetes or plasma glucose > 126 mg/dL, with anyone form of secondary arterial hypertension, renal transplant recipients, patients with hereditary or congenital kidney diseases, nephritic diseases, and known proteinuria/hematuria or poor-quality renal ultrasound recordings.

Patients with a history or clinical signs of heart failure (NYHA class II–IV), coronary artery disease, cerebrovascular disease, or major non-cardiovascular diseases (liver cirrhosis, obesity, chronic obstructive pulmonary disease, malignancies) were also excluded. Moreover, patients that refused to give consent and with missing data were also excluded.

### 4.4. Definition of Hypertensive Nephropathy

Hypertensive nephropathy occurs when chronic hypertension damages small vessels, glomeruli, and the tubulointerstitial compartment, leading to progressive kidney failure.

Hypertensive nephropathy progresses to kidney failure (formerly termed end-stage renal disease) in only a small proportion of patients. However, because chronic hypertension and hypertensive nephrosclerosis are common, hypertensive nephropathy is among one of the most frequent diagnoses in individuals with kidney failure.

The diagnosis is considered when laboratory studies indicate worsening renal function (rising serum creatinine and reduced eGFR, elevated blood urea nitrogen, and electrolyte and acid–base disturbances) in a patient with hypertension. The diagnosis rests on the medical history and on evidence of hypertension-related target-organ damage at physical examination (e.g., retinal changes, left ventricular hypertrophy). Hypertension must be present before the onset of proteinuria and kidney failure, and no other clinically suspected causes of kidney failure should be present.

In the present study, kidney biopsy was not performed as part of the protocol; therefore, the diagnosis relied on clinical criteria and exclusion of alternative etiologies based on history, laboratory evaluation, and imaging.

### 4.5. Med-Diet Definition and Classification

At enrolment, Mediterranean diet adherence was evaluated according to a PREDIMED questionnaire [21,22,23]. The PREDIMED questionnaire [8] consists of 14 items, each focusing on a specific aspect of the Mediterranean diet such as the use of olive oil, daily intake of fruits, vegetables, and legumes, fish, meats, nuts, whole grains, and red wine.

The total score ranges from 0 to 14, allowing classification of adherence to the diet as follows: low adherence (0–5 points), moderate adherence (6–9 points), and high adherence (10–14 points).

### 4.6. Clinical and Laboratory Measurement

During the initial clinical examination, a comprehensive medical history was obtained, including comorbidities and cardiovascular and renal risk factors such as arterial hypertension, diabetes mellitus, heart failure, and metabolic syndrome. Anthropometric measurements were recorded, and routine blood tests were performed, including a complete blood count, creatinine, urea, uric acid, lipid profile, and serum electrolytes (sodium, potassium, calcium, phosphorus, and magnesium), as well as parathyroid hormone and vitamin D levels. Additionally, patients’ smoking habits and current pharmacological treatments were documented.

### 4.7. OxS Measurement

To analyze OxS, blood samples were taken from our patients in fasting conditions. Blood was centrifuged for 10 min at 300 g at room temperature to obtain serum. Samples were immediately stored at −80 °C for analysis.

NOX2 is a isoform of the NADPH oxidase complex, a family of enzymes producing ROS [24]. To evaluate NOX2 activity, we measured serum levels of soluble NOX2-derived peptide (sNOX2-dp), a marker of NOX2 activation, by ELISA method as previously described [25]. Values were expressed as pg/mL; intra and inter-assay coefficients of variation were 5.2% and 6%, respectively.

Hydrogen peroxide (H_2_O_2_) in serum was evaluated by a Colorimetric Detection Kit (Arbor Assays, Ann Arbor, MI, USA). In brief, samples were mixed with a colorless colorimetric substrate and the reaction initiated by addition of horseradish peroxidase (HRP). The reaction is incubated at room temperature for 15 min. The HRP reacts with the substrate in the presence of H_2_O_2_ to convert the colorless substrate into a pink-colored product. The pink product was read at 560 nm. Values were expressed as μmol/L. Intra- and inter-assay coefficients of variation were 2.1% and 3.7%, respectively.

NO Due to the intrinsic instability and brief half-life of nitric oxide (NO)—approximately six seconds—its measurement was indirectly performed by quantifying stable metabolic by-products, nitrite (NO_2_^−^) and nitrate (NO_3_^−^), collectively referred to as NO [26]. NO levels were assessed in serum samples using a colorimetric assay kit (Abcam, Cambridge, UK). A volume of 100 µL of sample was incubated under constant stirring at 37 °C for 10 min. Concentrations were expressed in µM. The intra- and inter-assay coefficients of variation were 2.9% and 1.7%, respectively.

### 4.8. Statistical Analysis

Categorical variables were reported as numbers and percentages which were compared with Pearson’s χ2 test. Mean and standard deviation (SD) or median and interquartile range (IQR) were used for continuous variables, which were compared by Student’s *t*-test or Mann–Whitney U test, respectively. Normal distribution of variables was checked by the Kolmogorov–Smirnov test. We used Student unpaired *t* test and Pearson product-moment correlation analysis to evaluate normally distributed continuous variables and an appropriate nonparametric test (Mann–Whitney U test and Spearman rank correlation test) for the other variables. Group comparisons were performed using Fisher’s F-test (ANOVA) or Kruskal–Wallis test when needed. Clinical and laboratory characteristics were described according to RRI percentiles. Correlations between variables were assessed via Pearson’s or Spearman’s correlation coefficients, based on data distribution. Bonferroni test for multiple comparison was performed.

Finally, we performed a multivariable stepwise linear regression analysis evaluating the association between RRI and sNOX2-dp, NO and H_2_O_2_, respectively, adjusting for age, sex and concomitant use of beta blockers and statins that could influence OxS biomarkers.

All tests were 2-tailed and only *p*-values < 0.05 were considered statistically significant. The analyses were performed using SPSS 25.0 software (IBM, Armonk, NY, US).

## Figures and Tables

**Table 1:** Population characteristics according to renal resistive index (RRI) percentiles.

	<75th RRI (N:71)	≥75th RRI (N:28)	Total (N:99)	*p*-Value
*Age (years)*	59 [50, 65]	65 [61, 69]	60 [52, 66]	<0.001
*BMI*	25.7 ± 3.5	27.0 ± 2.7	26.0 ± 3.3	0.079
*sBP (mmHg)*	130 [120, 135]	130 [121, 140]	130 [120, 140]	0.259
*dBP (mmHg)*	80 [75, 85]	80 [75, 85]	80 [75, 85]	0.872
*mBP (mmHg)*	97 [90, 103]	97 [91, 101]	97 [90, 102]	0.797
*eGFR (ml/min/1.73 m^2^)*	79.1 ± 19.4	69.9 ± 21.0	76.5 ± 20.2	0.039
*Serum creatinine (mg(dL)*	0.98 ± 0.04	1.09 ± 0.08	1.01 ± 0.03	0.162
*Women (%)*	33 (46.5)	14 (50.0)	47 (47.5)	0.752
*Smoking (%)*	12 (16.9)	6 (21.4)	18 (18.2)	0.867
*Dyslipidemia (%)*	30 (42.3)	19 (67.9)	49 (49.5)	0.022
**Therapy**				
*ACE-I/ARBs (%)*	58 (81.7)	25 (89.3)	83 (83.8)	0.355
*Calcium channel blockers (%)*	38 (53.5)	11 (39.3)	49 (49.5)	0.202
*β-blockers (%)*	19 (26.8)	18 (64.3)	37 (37.4)	0.001
*Statin (%)*	17 (23.9)	16 (57.1)	33 (33.3)	0.002
*α -blockers (%)*	2 (2.8)	0 (0.0)	2 (2.0)	0.370
*Diuretics (%)*	16 (22.5)	8 (28.6)	24 (24.2)	0.528
**Biomarkers**				
*Nitric oxide (µM)*	14.6 [11.2, 19.0]	11.4 [6.2, 13.9]	13.4 [10.5, 18.7]	0.001
*sNOX2-dp (pg/mL)*	19.0 [16.4, 27.7]	19.6 [15.8, 32.5]	19.0 [16.3, 29.0]	0.706
*Hydrogen peroxide (µmol/L)*	14.4 [11.9, 19.2]	18.2 [13.8, 22.7]	15.1 [12.0, 20.7]	0.011
**Med-Diet adherence**				
*Med-Diet score*	8 [7, 9]	7 [5, 9]	8 [6, 9]	0.160
*Low (%)*	8 (11.3)	7 (25.0)	15 (15.2)	0.155
*Moderate (%)*	48 (67.6)	18 (64.3)	66 (66.7)	
*High(%)*	15 (21.1)	3 (10.7)	18 (18.2)	

ACE-I/ARBs: angiotensin converting enzyme inhibitor/angiotensin receptor blockers, BMI: body mass index, dBP: diastolic blood pressure, eGFR: estimated glomerular filtration rate, mBP: mean blood pressure, sBP: systolic blood pressure.

**Table 2:** Multivariable stepwise linear regression analysis evaluating association between renal resistive index (RRI) and oxidative stress biomarkers.

Multivariable *	Beta	B	95% Confidence Interval	*p*-Value
RRI and sNOX2-dp	0.310	39.0	14.9 to 63.1	0.002
RRI and H_2_O_2_	0.305	24.8	9.2 to 40.5	0.002
RRI and NO	−0.248	−25.0	−44.7 to −5.3	0.013

* Adjusted for age, sex, beta-blockers, and statin use. H_2_O_2_: hydrogen peroxide, NO: nitric oxide, sNOX2-dp: soluble NOX2-derived peptide.

## Data Availability

The data presented in this study are available on request from the corresponding author. The data are not publicly available due to privacy or ethical restrictions.
